# Mumblety-Peg: a potential cause of flesh wounds and ocular trauma

**DOI:** 10.5249/jivr.v7i2.529

**Published:** 2015-07

**Authors:** Anand N. Bosmia, Christoph J. Griessenauer, R. Shane Tubbs

**Affiliations:** ^*a*^Children’s of Alabama – Pediatric Neurosurgery Department, Ambulatory Care Center, Birmingham, Alabama.; ^*b*^ Department of Surgery, Division of Neurosurgery, The University of Alabama, Birmingham, Alabama.

The game of mumblety-peg, which involves performing various maneuvers with a pocketknife, has been present in America since at least the nineteenth century^1,2^ and was prevalent among school-aged children, cowboys, and soldiers who fought in World War I and World War II.^2^ Its popularity waned in the 1970’s as adults began to ban the game at summer camps and carrying a pocketknife became less prevalent among American males.^2^ However, per online parental resources published this year, mumblety-peg is becoming more popular among children and adolescents.^3,4^ Primary care physicians and emergency physicians may encounter individuals, particularly adolescent males, who present with injuries secondary to participating in this game.

In one version of the game, two participants take turns throwing a pocketknife at the ground, and the player whose knife sticks in the ground closer to his own foot is the winner. ^2^ Another version involves tossing the knife in progressively difficult ways with the aim of getting the knife to stick in the ground. ^2^ The concluding act of this version gives mumblety-peg its name. The first person who completes the tosses hammers a wooden peg into the ground with the handle of his knife. The last person to complete the tosses must pull the peg out of the ground with his teeth, and as this individual likely will “mumble” curses at the winner, the game was named “mumblety-peg”.^2^ Online parental resources describe a version of mumblety-peg in which the participant spreads his fingers on a table or his toes on the ground and then stabs at the interdigital spaces as quickly as possible while avoiding cutting himself.^3,4^

The rituals of mumblety-peg predispose its participants to various injuries. The risk of stabbing wounds is high.^3^ In some versions of the game, a player can automatically win if he purposely throws his knife into his own foot.^2^ Participants are especially at risk for lower extremity injuries if they play the game barefooted. As mumblety-peg is more likely to be played outdoors, flesh wounds may be exposed to soil and thereby inoculated with bacteria. Also, ocular trauma may result from playing mumblety-peg. In one case, a ten-year-old boy was pushed by a playmate such that his face landed on the point of his knife, which penetrated his right eye and caused an iris hernia.^5^

In conclusion, primary care physicians and emergency physicians may encounter patients who present with injuries secondary to playing mumblety-peg. Ophthalmologists and infectious disease specialists may become involved in the care of patients who sustain ocular trauma from playing mumblety-peg or whose wound sites become infected.
